# Adolescent conduct problems and premature mortality: follow-up to age 65 years in a national birth cohort

**DOI:** 10.1017/S0033291713001402

**Published:** 2013-08-21

**Authors:** B. Maughan, M. Stafford, I. Shah, D. Kuh

**Affiliations:** 1 MRC Social, Genetic and Developmental Psychiatry Centre at King's College London Institute of Psychiatry, London, UK; 2 MRC Unit for Lifelong Health and Ageing, London, UK

**Keywords:** Conduct problems, life course, mortality, National Survey of Health and Development

## Abstract

**Background:**

Severe youth antisocial behaviour has been associated with increased risk of premature mortality in high-risk samples for many years, and some evidence now points to similar effects in representative samples. We set out to assess the prospective association between adolescent conduct problems and premature mortality in a population-based sample of men and women followed to the age of 65 years.

**Method:**

A total of 4158 members of the Medical Research Council National Survey of Health and Development (the British 1946 birth cohort) were assessed for conduct problems at the ages of 13 and 15 years. Follow-up to the age of 65 years via the UK National Health Service Central Register provided data on date and cause of death.

**Results:**

Dimensional measures of teacher-rated adolescent conduct problems were associated with increased hazards of death from cardiovascular disease by the age of 65 years in men [hazard ratio (HR) 1.17, 95% confidence interval (CI) 1.04–1.32], and of all-cause and cancer mortality by the age of 65 years in women (all-cause HR 1.16, 95% CI 1.07–1.25). Adjustment for childhood cognition and family social class did little to attenuate these risks. Adolescent conduct problems were not associated with increased risks of unnatural/substance-related deaths in men or women in this representative sample.

**Conclusions:**

Whereas previous studies of high-risk delinquent or offender samples have highlighted increased risks of unnatural and alcohol- or substance abuse-related deaths in early adulthood, we found marked differences in mortality risk from other causes emerging later in the life course among women as well as men.

## Introduction

More than half a century ago now, Robins' classic follow-up of child guidance attenders provided the first evidence that the many adverse outcomes of childhood conduct problems included the most adverse outcome of all: an increased risk of early death (Robins & O'Neal, 1958; Robins, 1966). Since that time, numerous other reports have confirmed that highly delinquent young men are at increased risk of death in early adulthood as a result of accidents, homicide, suicide and the effects of alcoholism (for a review, see Piquero *et al.* 2011). Much of this increased risk appears to reflect relatively direct effects of involvement in impulsive, delinquent and dangerous behaviours.

Subsequent research has extended this picture in a variety of ways. First, follow-ups of population samples have shown that severe and/or persistent childhood and adolescent antisocial behaviour is associated not only with increased exposure to injuries and accidents in early adulthood (Farrington, 1995) but also with a range of other health-related risks (Odgers *et al.* 2007; Piquero *et al.* 2007; Colman *et al.* 2009). In the Dunedin Multidisciplinary Health and Development Study, for example, Odgers *et al.* (2007) found that at the age of 32 years ‘life-course persistent’ males (men showing early-onset and persisting antisocial behaviour) reported themselves in poorer general health than men on a low antisocial trajectory; were more likely to have consulted their general practitioner (GP) and been hospitalized in the past year; were markedly more likely to be smokers; had increased risk of gum disease and symptoms of chronic bronchitis; and had elevated levels of high-sensitivity C-reactive protein, a marker of cardiovascular risk. Though the great majority of research in this area has focused on men, where samples of young women have been studied, evidence suggests that their physical health may also be compromised (see, for example, Odgers *et al.* 2008). Findings of this kind suggest that highly antisocial individuals may continue to be at increased risk of premature mortality later in the life course, but through somewhat different pathways from those contributing to their early risk of violent or unnatural death.

Evidence is now accumulating to support that view. Laub & Vaillant (2000), for example, undertook a long-term follow-up of death rates in the Gluecks’ sample of court-adjudicated delinquent and non-delinquent boys (Glueck & Glueck, 1950). By the age of 65 years, 42% of the delinquents were known to have died, by comparison with only 27% of the non-delinquents; group differences in death rates were significant before the age of 40 years, but became if anything more marked in later middle age, largely as a result of increased mortality from natural causes in the delinquent group between the ages of 40 and 65 years (27% *versus* 19%). The leading cause of death in both groups was heart disease, but it was more frequent among delinquent subjects. Severity of juvenile antisocial behaviour, alcohol abuse, adult criminal behaviour, dysfunctional upbringing and poor education all showed modest bivariate associations with premature death; only juvenile antisocial behaviour and alcohol abuse remained significant predictors in a multivariate model. As a result, Laub & Vaillant (2000) speculated that unhealthy adult life-styles, rather than more distal family and social conditions in childhood, might constitute the key risk pathways.

The Gluecks drew their sample from inner-city Boston, and their delinquent boys had histories of persistent delinquency, serious enough to have required placement in correctional institutions. Though evidence from long-term follow-ups of more representative samples remains limited, such findings as are available (see, for example, Trumbetta *et al.* 2010; Piquero *et al.* 2011) also point to an increased mortality risk among offenders. In addition, Jokela *et al.* (2009) have recently reported increased mortality associated with much less severe markers of childhood antisocial behaviour in men and women from a nationally representative sample. Studying death rates in the 1958 British birth cohort (the National Child Development Study), they found that teacher ratings of broadly ‘externalizing’ behaviours at the ages of 7 and 11 years were consistent predictors of mortality between the ages of 14 and 46 years. As expected, death rates were relatively low in these ages; nonetheless, a 1-s.d. increase on the teacher behaviour ratings was associated with a 27% increased relative risk of mortality (from 2.1% to 2.7%). Controls for childhood cognitive ability, social class, family size and family difficulties attenuated this effect somewhat (to 19%), but it remained highly significant, and as salient in women as in men.

Jokela *et al.* (2009) had no data on causes of death, limiting their capacity to examine possible contributory pathways. Commenting on their findings, however, Angold (2009) suggested a number of potential mechanisms, including: (i) direct results of psychopathology (such as death by drug overdose); (ii) secondary effects of psychopathology, such as deaths by accident or homicide, or from the health effects of chronic alcohol and drug (including tobacco) abuse; (iii) deaths from physical diseases known to be associated with psychiatric disorders, including, in the present context, known links between cardiovascular morbidity and hostility; (iv) effects driven by the environmental correlates of psychopathology, such as low social status, involvement in relatively dangerous jobs, or restricted access to good-quality housing and nutrition; (v) genetic and early-life (including intrauterine) factors that may predispose both to psychiatric and physical disorders; and (vi) iatrogenic fatalities, related to treatments for psychiatric conditions or their physical disorder concomitants.

We report here on associations between adolescent antisocial behaviour and premature mortality in the 1946 British birth cohort, the Medical Research Council National Survey of Health and Development (NSHD). Like the National Child Development Study, the NSHD is a nationally representative birth cohort study, but with a longer follow-up, to the age of 65 years. Previous analyses of the NSHD have shown that teacher-rated adolescent conduct problems predicted a wide spectrum of adverse outcomes (including poor mental health, problems in family life and relationships, and poor educational and economic progress) throughout adult life (Colman *et al.* 2009). We hypothesized that teacher-rated conduct problems would also predict premature mortality.

## Method

### Sample

The Medical Research Council's NSHD is a prospective national birth cohort of 5362 individuals, a socially stratified sample of all births that took place in England, Scotland and Wales during 1 week in March 1946. The whole sample was studied on 17 occasions up to the age of 26 years, and has since been evaluated at the ages of 31, 36, 43, 53 and 60–64 years. Comparisons with census data show that the remaining cohort is broadly representative of all native-born adults currently resident in England, Scotland and Wales (Wadsworth *et al.* 2003; Stafford *et al.* 2013).

### Adolescent conduct problems

Full details of the measure of adolescent conduct problems have been presented elsewhere (Colman *et al.* 2009). In brief, a range of behaviours was assessed by teachers at the ages of 13 and 15 years, using questionnaires that were forerunners of the Rutter behaviour rating scales. Individual items of behaviour were rated as occurring more frequently than, at the same rate as, or less frequently than for other children in the class. Confirmatory factor analysis for categorical data (normal ogive item response models) identified a seven-item ‘conduct problems’ factor that included items indexing disobedience, lying, lack of punctuality, restlessness, truancy, day dreaming in class, and poor response to discipline.

Scores for these seven items were summed to create scales (*α* = 0.69 at age 13 years, *α* = 0.75 at age 15 years, possible range 0–14); see online Supplementary Fig. 1*a,b* for distributions. Scores at these two ages were moderately correlated (*ρ* = 0.50). We computed mean conduct problem scores from the ages of 13 and 15 years for cases where both measures were available (*n* = 3651), and used the available single-age measures for the remainder of the sample (*n* = 550). Although data on antisocial behaviour at earlier ages were limited, young people identified as being in the highest quartile of adolescent conduct problems were more likely to have been labelled aggressive at the age of 10 years by both their teachers [odds ratio (OR) 11.36, 95% confidence interval (CI) 5.16–25.28] and their mothers (OR 3.20, 95% CI 2.01–5.09). We used the conduct problem scores as dimensional predictors of mortality risk, as well as examining predictions for young people in the top 5% *versus* the remaining 95% of the conduct problems range.

**Fig. 1.**
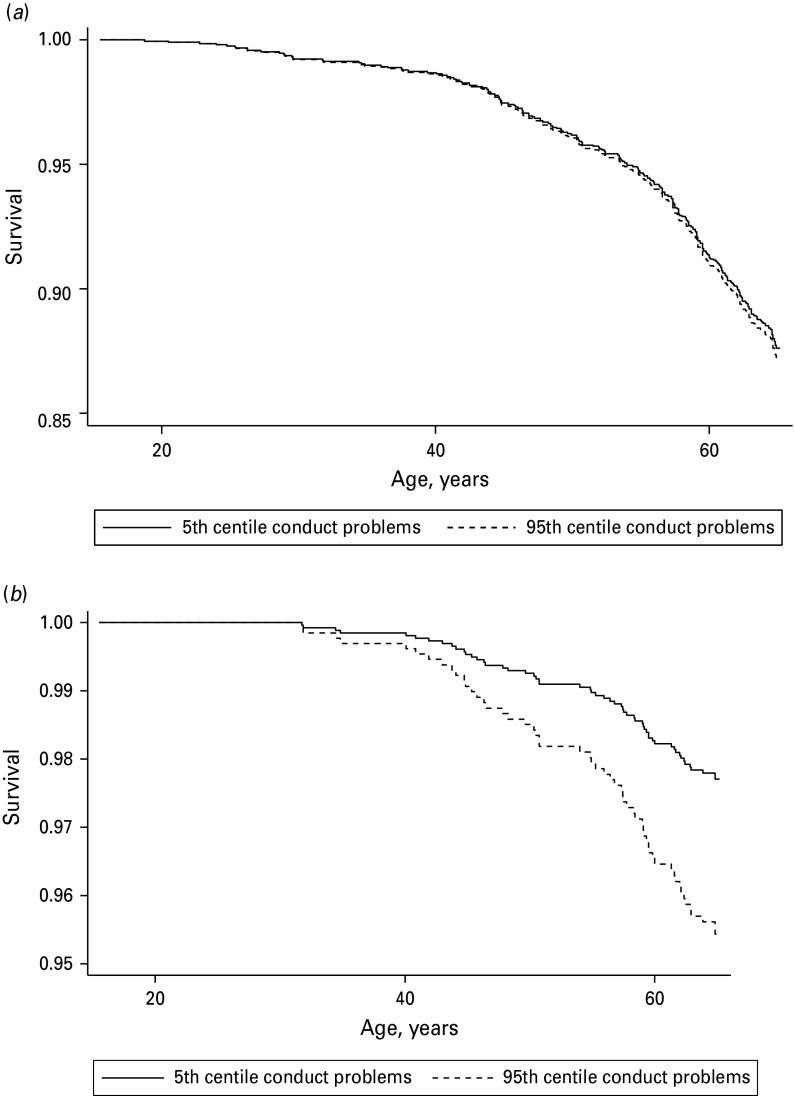
(*a*) Predicted all-cause survival probability adjusted for childhood social class and cognition: based on 242 deaths in 2103 men. (*b*) Predicted coronary heart disease survival probability adjusted for childhood social class and cognition: based on 56 deaths in 2103 men. Cox proportional hazards regressions adjusted for childhood social class and cognition.

At the age of 26 years, NSHD study members were linked to the National Health Service Central Register which provides a reliable indicator of all deaths in the UK, along with a record of cause of death. The follow-up period covered in the present analyses continued from the end of data collection at the age of 15 years until the first of death, emigration, or the end of March 2011, the cohort members’ 65th birthday. Data for study members who emigrated during the follow-up were censored at the mid-year of the next data collection following emigration.

The underlying cause of death was coded using either International Classification of Diseases, Ninth Revision (ICD-9) or Tenth Revision (ICD-10) disease classifications according to standard rules. We focus here on deaths from: (i) cardiovascular diseases (ICD-9 codes 401–454 and ICD-10 codes 110–189); (ii) cancers (ICD-9 codes 140–239 and ICD-10 codes C00–C97); and (iii) unnatural causes, including alcohol/drug abuse and associated liver disease (ICD-9 codes 291–292, 295–305, 307–309, 311–316, 570–571 and ICD-10 codes F61–F69, K70–K71) along with accidents, assaults and self-injurious behaviour (ICD-9 codes 800–994, 1800–1869, 1880–1999 and ICD-10 codes S00–X99).

### Possible confounding factors

Childhood factors previously found to be associated with premature adult mortality in this cohort, namely social class of origin and childhood cognitive ability (Kuh *et al.* 2009*b*), were chosen *a priori* as potential confounders. Social class of origin was based on father's occupation at the age of 4 years classified according to the Registrar General's 1971 classification, and dichotomized to manual and non-manual (the reference group). Father's occupation at the age of 7 years was used if father's occupation at the age of 4 years was missing. Childhood cognitive ability was measured at the ages of 8, 11 and 15 years using tests designed by the National Foundation of Education Research and described in detail elsewhere (Richards *et al.* 2004; Kuh *et al.* 2009*a*). The total scores at each age, obtained by standardizing the sum of individual test scores, were highly correlated (*ρ* = 0.81 for scores at the ages of 8 and 11 years and *ρ* = 0.88 for scores at the ages of 11 and 15 years; Kuh *et al.* 2009*a*). In this analysis, we used the score at the age of 11 years where available, or at the age of 8 years (or age 15 years) if not.

### Missing data

Of the original birth cohort, 252 died before the age of 16 years, 912 had missing data on adolescent conduct problems and 40 either did not consent to National Health Service mortality flagging or had a missing date of death, giving data on 4158 study members in the analytic sample. A further 101 cases had missing data on childhood social class or cognition, giving data on 4057 study members for fully adjusted analyses of all-cause mortality. Compared with the sample with complete data, excluded study members were more likely to come from a non-manual social class of origin, but there was no evidence of differences in cognitive abilities.

### Statistical methods

For descriptive purposes, study members were grouped into quartiles on the basis of their conduct problems score (cut-points at 0.5, 1.5 and 2.5). Cox's proportional hazards models were then used to investigate relationships between dimensional indicators of adolescent conduct problems and survival time. The first set of models examined all-cause mortality and cause-specific mortality without covariate adjustment. The second set of models adjusted for childhood social class and cognition. No evidence against the assumption of proportional hazards was found in any of the models. We additionally tested an accelerated failure time model to assess whether associations between conduct problems and survival time varied across the period of the follow-up. Because previous work in this cohort indicates some gender differences in the associations between childhood factors and later outcomes (Kuh *et al.* 2009*b*; Strand *et al.* 2012), we conducted gender-specific analyses and examined the interactions of conduct problems with gender. In sensitivity analysis, we replicated the second set of models for the adjusted association between conduct problems and all-cause mortality examined for the subset of study members who had two measures of adolescent conduct problems. All analyses were conducted using Stata SE v. 12 (StataCorp LP, USA).

## Results

### Sample characteristics

Characteristics of the analytic sample are summarized in Table 1. As expected, rates of adolescent conduct problems were relatively low in this general population sample, and boys had somewhat higher scores than girls (median = 1 and 1.5, respectively). Adolescent conduct problems were associated with both of the selected childhood confounders. Conduct problem scores were negatively correlated with childhood cognition (*ρ* = –0.28), and girls and boys from manual social class backgrounds were more likely to be rated as scoring in the top quartile of the conduct problems scale (22.5% of girls and 30.0% of boys) than those from non-manual social class backgrounds (15.3% of girls and 19.1% of boys).

**Table 1.** Sample characteristics

	Total sample (*n* = 4158)	Men (*n* = 2164)	Women (*n* = 1994)
Adolescent conduct problems^a^			
Median (IQR)	1.5 (2.0)	1.5 (2.5)	1.0 (1.5)
Childhood confounders			
Childhood social class, *n* (%)			
Non-manual	1602 (38.5)	828 (38.3)	774 (38.8)
Manual	2461 (59.2)	1278 (59.1)	1183 (59.3)
No father/not working	59 (1.4)	32 (1.5)	27 (1.4)
Missing	36 (0.9)	26 (1.2)	10 (0.5)
Deaths ages 16–65 years, *n* (%)			
All-cause mortality	422 (10.1)	250 (11.6)	172 (8.6)
Cause-specific mortality, *n* (% all deaths)			
Cancers	174 (41.2)	93 (37.2)	81 (47.1)
Coronary heart disease	93 (22.0)	57 (22.8)	36 (20.9)
Unnatural causes^b^	60 (14.2)	40 (16.0)	20 (11.6)
Other causes	95 (22.5)	60 (24.0)	35 (20.3)

IQR, Interquartile range.

a Possible range 0–14.

b Unnatural causes: alcohol/drug abuse and associated liver disease, accidents, assaults and self-injurious behaviour.

Table 1 also shows descriptive data on mortality rates in the cohort between the ages of 16 and 65 years. Approximately one in 12 women and one in eight men were known to have died during the follow-up period. Cancer was the leading case of death in both genders, accounting for over a third of deaths in men and approaching half of those in women. Around one in five deaths were associated with coronary heart disease in both genders, while deaths from unnatural causes were less common (16.0% men, 11.6% women). All-cause mortality was associated with both childhood confounders. The unadjusted hazard ratio associated with manual childhood social class was 1.85 (95% CI 1.30–2.61) in women and 1.23 (95% CI 0.94–1.62) in men. The unadjusted hazard ratio associated with each unit increase in childhood cognition was 0.84 (95% CI 0.71–0.99) in women and 0.80 (95% CI 0.69–0.91) in men.

### Adolescent conduct problems and mortality

Table 2 shows hazard ratios and 95% CIs for associations between adolescent conduct problems and survival time in relation to all-cause mortality and the three cause-specific mortality categories for men and for women. All-cause mortality was unrelated to adolescent conduct problems in men, as were deaths from cancers and unnatural causes; the unadjusted hazard of dying from coronary heart disease, however, increased by 17% for each unit increase in conduct problems. There was no evidence that the magnitude of the association between conduct problems and hazard varied across the follow-up period, nor of non-linearity in the association between conduct problems and mortality (suggesting a linear increase in risk with increasing severity of conduct problems). The adjusted hazard ratio for death from coronary heart disease was 1.14 (95% CI 1.00–1.29), indicating a small degree of attenuation on adjustment for other childhood factors. To illustrate the effects for men with high levels of conduct problems, Fig. 1*a* (all-cause mortality) and Fig. 1*b* (coronary heart disease mortality) show predicted survival probabilities (adjusted for childhood social class and cognition) for men at the 5th and 95th centiles of the conduct problems distribution.

**Table 2.** Risk for all-cause and cause-specific mortality ages 15–65 years per 1-unit increase in adolescent conduct problems, obtained from Cox's proportional hazards models based on 4057 study members with valid childhood social class and cognition

Cause of death	Men (*n* = 2103)			Women (*n* = 1954)			Gender interaction: *p*
	Deaths, *n*	Unadjusted	Adjusted for childhood social class and cognition	Deaths, *n*	Unadjusted	Adjusted for childhood social class and cognition	
All-cause mortality	242	1.04 (0.98–1.12)	1.01 (0.94–1.08)	171	1.16 (1.07–1.25)***	1.13 (1.04–1.23)**	0.04
Cause-specific mortality							
Cancers	90	0.99 (0.88–1.11)	0.94 (0.83–1.07)	80	1.16 (1.03–1.30)*	1.15 (1.02–1.30)*	0.06
Coronary heart disease	56	1.17 (1.04–1.32)*	1.14 (1.00–1.29)	36	1.10 (0.92–1.32)	1.03 (0.85–1.25)	0.5
Unnatural causes	39	0.98 (0.82–1.18)	0.97 (0.80–1.17)	20	1.07 (0.84–1.37)	1.05 (0.81–1.36)	0.6

Data are given as hazard ratio (95% confidence interval).

* *p* < 0.05, ** *p* < 0.01, *** *p* < 0.001.

**Fig. 2.**
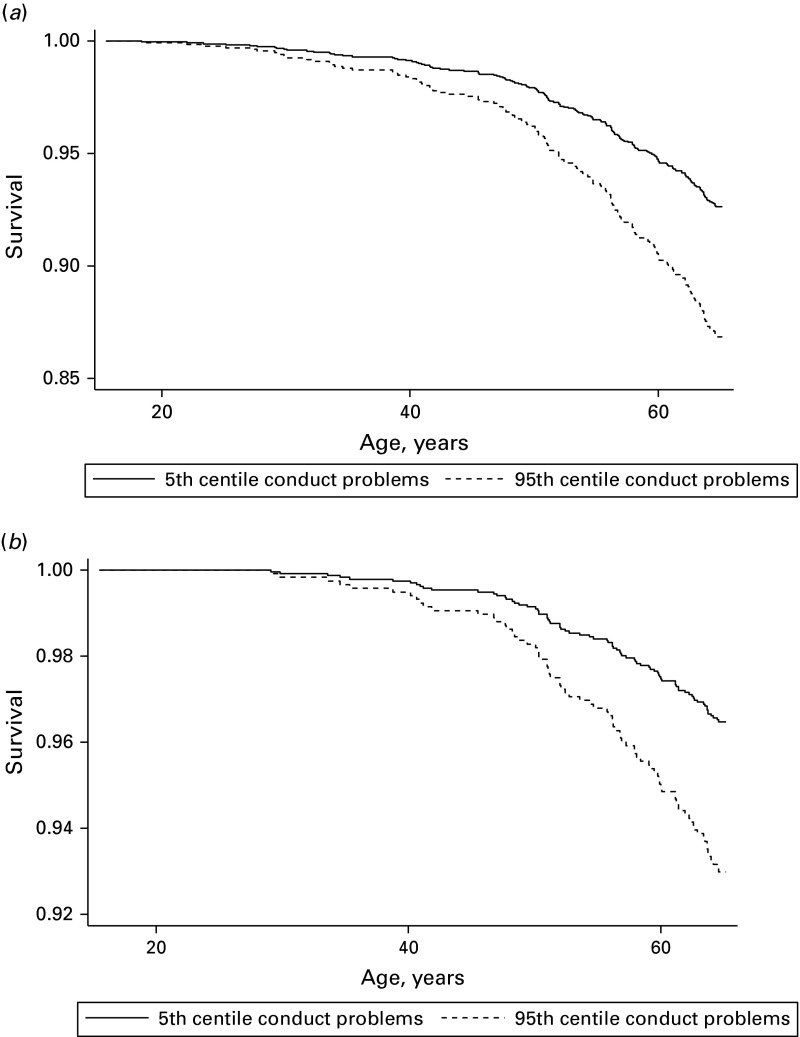
(*a*) Predicted all-cause survival probability adjusted for childhood social class and cognition: based on 171 deaths in 1954 women. (*b*) Predicted cancer survival probability adjusted for childhood social class and cognition: based on 80 deaths in 1954 women. Cox proportional hazards regression adjusted for childhood social class and cognition.

For women, each unit increase in adolescent conduct problems was associated with an increased hazard ratio for all-cause mortality by the age of 65 years, which remained significant after adjustment for childhood social class and cognition. As Table 2 shows, deaths from cancers appeared to be the most salient contributors to this increased mortality risk; once again, adjustment for childhood social class and cognition did not materially attenuate the associations. Fig. 2*a* (all-cause mortality) and Fig. 2*b* (cancer mortality) illustrate these effects for women at the 95th and 5th centiles of the conduct problems scale [all-cause mortality: adjusted hazard ratio 2.10 (95% CI 1.43–3.08); cancer mortality: adjusted hazard ratio 2.03 (95% CI 1.13–3.65)]. There was a slightly elevated hazard of death from coronary heart disease among women, although the CI was wide. There was no clear evidence of an association between conduct problems and deaths from unnatural causes.

Gender differences in associations with adolescent conduct problems were evident for all-cause mortality (*p* = 0.04) and cancers. The test for interaction between gender and conduct problems on coronary heart disease mortality did not approach statistical significance, but lack of statistical power to investigate coronary heart disease deaths, rather than lack of association, probably explains the somewhat different findings for men and women on this outcome.

There were no differences in adolescent conduct problems score or proportion dying during follow-up for men who had one compared with two measures of conduct problems. Women who only had one measure had a higher conduct problems score than women who had both measures. In all but one model, the association between conduct problems and mortality was closer to the null for those with one *versus* those with two measures of conduct problems though differences were very small in magnitude and did not affect the conclusions (data available on request). The exception was for cancer mortality among women, for whom the adjusted hazard ratio was 1.12 (95% CI 0.98–1.28) when only those with two measures of conduct problems were considered.

## Discussion

Our findings add to the growing (but still small) body of evidence that childhood and adolescent conduct problems are associated with an increased risk of premature mortality in representative, population-based samples as well as in the high-risk and offender samples studied in much past research. Tracking a nationally representative sample from adolescence to early old age, we found a dose–response relationship between severity of teacher-rated conduct problems and all-cause and cancer mortality in women, and between conduct problems and coronary heart disease mortality in men. Despite clear associations between social class of origin and childhood cognitive ability and both conduct problems and mortality, these early characteristics did not account for the increased mortality risk. In addition, we found such associations when using dimensional ratings of adolescent conduct problems, as well as categorically defined ‘high’ conduct problem scores, as the predictors in our analyses. The NSHD ratings of conduct problems were captured in late 1950s and early 1960s, and preceded the development of the major behaviour-rating scales in widespread use today. Nevertheless, the available measures allowed for the construction of scales with adequate reliability, good face validity, and known associations with other adverse adult outcomes highly consistent with findings from past follow-ups of conduct problem samples (Colman *et al.* 2009). Using these measures we not only found increased mortality risk for young people in the extremes of the conduct problems distribution, but also that that risk extended in a linear fashion across the range of teacher-rated conduct problem scores.

Where most past studies have focused only on men (Piquero *et al.* 2011), or failed to identify gender differences in the relationship between conduct problems and mortality (Jokela *et al.* 2009), our data also pointed to the possibility of gender-specific associations between adolescent conduct problems and subsequent mortality risk. As expected, levels of adolescent conduct problems were higher in males than in females, but associations with premature mortality were if anything stronger in women than in men. Past studies have documented robust associations between adolescent conduct problems and poor physical health in female samples in early adulthood (Bardone *et al.* 1998), and at mid-life (von Stumm *et al.* 2011), with some pointers in the latter study to differing patterns of association for women and men. In the NSHD women, associations between conduct problems and premature mortality were primarily driven by an increased likelihood of death from cancers, whereas among men conduct problems primarily related to deaths from coronary heart disease. The associations between adolescent conduct problems and mortality align with recent work in general population samples (Jokela *et al.* 2009) and long-term follow-ups of delinquent samples (Laub & Vaillant, 2000), suggesting that unhealthy adult social circumstances and life-styles might constitute key elements in the risk pathway. The specific adult factors associated with adolescent conduct problems may differ for men and women, although recent evidence from the NSHD found few clear pointers to effects of this kind. Colman *et al.* (2009), using data to the age of 53 years, found that symptoms of depression and anxiety, early parenthood, divorce, unhappiness with family life, problems in relationships with others, low educational attainment, low adult socio-economic position and financial difficulties were all more likely to be experienced by adolescents with severe conduct problems, but identified few gender differences in the relationship between conduct problems and this range of adult outcomes. In that study, gender interacted with conduct problems only for a self-reported history of nervous trouble. It remains possible, however, that the psychological, social and economic outcomes identified by Colman *et al.* relate differently to cause-specific mortality in men and women, highlighting important avenues for future research.

In contrast to past studies we found no associations between adolescent conduct problems and all-cause mortality in males, and no links with risk of unnatural deaths in women or in men. In part, these findings may reflect the nature of the conduct problem indicators available in the NSHD. As outlined earlier, the ratings focused primarily on indicators of non-aggressive, rather than aggressive conduct problems, and were made at the ages of 13 and 15 years. Moffitt (2003, 2006) has proposed that morbidity and mortality risks are most likely to be elevated in groups with early (i.e. childhood)-onset conduct problems where difficulties persist to adolescence and beyond. The few direct tests of Moffitt's predictions to date (Odgers *et al.* 2008; Piquero *et al.* 2011) have confirmed poorer outcomes in early-onset/persistent groups, but have also shown some elevated risk associated with (typically less severe) antisocial behaviour beginning in adolescence. Our adolescent ratings showed strong associations with indicators of both teacher- and parent-rated aggression at the age of 10 years, but will inevitably tap both childhood- and adolescent-onset conduct problems. As a result, our findings are likely to give conservative estimates of mortality risks associated with childhood-onset antisocial behaviour, but may well overestimate risks for truly ‘adolescence limited’ youth. It is also possible, however, that the premature mortality from accidents, suicide and alcohol-related difficulties reported in many high-risk delinquent and offender samples are indeed less common sequelae of conduct problems in more normative samples. Replications in other population-based samples are needed to test this out.

Our study was based on data from a nationally representative sample of women and men followed from adolescence to the age of 65 years, using all-cause and cause-specific mortality data confirmed independently for all except migrant study members. The analyses are based on the 79% of the original cohort who survived to the age of 15 years (when conduct problems were assessed). Those excluded because of missing data were more likely to have fathers in higher social classes but more likely to be materially disadvantaged on other indicators (such as crowding) and did not differ from the analysis sample on cognition in childhood. As outlined earlier, the main limitation of the NSHD dataset for analyses of this kind is that the available measures of conduct problems focused on relatively mild indicators of behavioural difficulties, assessed in the early and mid-teens. As a result, our findings may underestimate the mortality risk associated with early-onset and/or aggressive conduct problems in community samples. By the same token, however, the findings also suggest that even relatively mild adolescent behaviour problems may carry implications for individuals’ later physical health. It may also be relevant to note here that studies of subsequent UK birth cohorts have identified rising levels of adolescent conduct problems over the last quarter of the twentieth century (Collishaw *et al*. 2004), suggesting that this burden is unlikely to have attenuated in more recent years.

Our analyses cannot provide direct pointers to the causal pathways involved here. Of the variety of mechanisms that has been postulated (Angold, 2009), however, the lack of associations between adolescent conduct problems and deaths from unnatural causes in our sample suggests that the direct effects of dangerous or impulsive behaviours may be less salient in representative samples than the long-term impact of childhood behaviour problems on adult social conditions, patterns of self-care and health-related life-style factors (von Stumm *et al*. 2011). In addition, processes whereby chronic stress exposure early in development contribute to long-term health susceptibilities via allostatic overload (see, for example, Evans, 2003), inflammation (Odgers *et al.* 2007) or a dysregulated hypothalamic–pituitary–adrenal axis (Pajer *et al.* 2001; Fairchild *et al.* 2008; Pesonen *et al.* 2011) may also be relevant here. Further studies of these differing possibilities are clearly needed to guide intervention strategies.

In summary, conduct problems identified in adolescence predict higher relative risk of premature death to the age of 65 years, with deaths from cancer the main contributor to this association in women, and deaths from coronary heart disease the main contributors in men. Our findings provide support for the hypothesis that, in addition to excess deaths from violent and unnatural causes in early adulthood, adolescent conduct problems have persistent effects even into early older age and reinforce calls for greater investment in early prevention and intervention efforts to reduce conduct problems in childhood and adolescence (Richards *et al.* 2009; Allen, 2011). Conduct problems have only rarely been studied as early determinants of coronary heart disease and cancers. Whilst socio-economic factors, health behaviours, depression and anxiety disorders, inflammation and hypothalamic–pituitary–adrenal axis dysregulation have been linked to these diseases and to conduct problems in separate studies, further research is warranted to explicitly investigate their role and other potential causal processes.

## Supplementary Material

Supplementary Material Supplementary information supplied by authors.

## Supplementary material

For supplementary material accompanying this paper visit http://dx.doi.org/10.1017/S0033291713001402.
