# Supplementary material for: Adolescent conduct problems and premature mortality: follow-up to age 65 years in a national birth cohort
Source: Psychol Med. 2013 Aug 21;44(5):1077–86. doi: 10.1017/S0033291713001402 (PMC3948505; doi:10.1017/S0033291713001402)
Supplement: Supplementary Material — Supplementary information supplied by authors. [file S0033291713001402sup001.doc]

**Supplementary Fig. S1.** Distribution of NSHD conduct problems score at 13-15 years for (*a*) male study members and (*b*) female study members

(*a*)

(*b*)
